# Attenuating Cardiac Pulsations within the Cochlea: Structure and Function of Tortuous Vessels Feeding Stria Vascularis

**DOI:** 10.1155/2013/941757

**Published:** 2013-05-19

**Authors:** Mattia Carraro, Jaina Negandhi, Jafri Kuthubutheen, Evan J. Propst, Lukas Kus, Vincent Y. W. Lin, Robert V. Harrison

**Affiliations:** ^1^Auditory Science Laboratory, Department of Otolaryngology, and Program in Neuroscience and Mental Health, The Hospital for Sick Children, Toronto, ON, Canada M5G 1X8; ^2^Institute of Biomaterials and Biomedical Engineering, University of Toronto, Toronto, ON, Canada M5S 1A1; ^3^Sunnybrook Research Institute, Sunnybrook Health Science Centre 2075 Bayview Avenue, Toronto, ON, Canada M4N 3M5; ^4^Department of Otolaryngology-Head and Neck Surgery, University of Toronto, Toronto, ON, Canada M5S 1A1

## Abstract

The mammalian ear has an extraordinary capacity to detect very low-level acoustic signals from the environment. Sound pressures as low as a few **μ**Pa (−10 dB SPL) can activate cochlear hair cells. To achieve this sensitivity, biological noise has to be minimized including that generated by cardiovascular pulsation. Generally, cardiac pressure changes are transmitted to most peripheral capillary beds; however, such signals within the stria vascularis of the cochlea would be highly disruptive. Not least, it would result in a constant auditory sensation of heartbeat. We investigate special adaptations in cochlear vasculature that serve to attenuate cardiac pulse signals. We describe the structure of tortuous arterioles that feed stria vascularis as seen in corrosion casts of the cochlea. We provide a mathematical model to explain the role of this unique vascular anatomy in dampening pulsatile blood flow to the stria vascularis.

## 1. Introduction

Without a stethoscope, we do not normally hear our own heart beat, although there are some pathological conditions when it can be heard (e.g., high blood pressure, pulsatile tinnitus, and superior semicircular canal dehiscence). In these cases, the perceived heart sound is usually the result of signals from arteries external to the cochlea rather than pressure changes generated within cochlear vessels. The energy requirements of the cochlea are high, in particular the for Na/K ion exchange pumps that generate the endolymphatic potential. A good vascular supply is essential but mechanical stimulation has to be minimized. The cochlea appears to have a unique blood supply vasculature that ensures a dampening of cardiovascular pressure changes, essentially converting a pulsatile flow to a more linear stream. The requirement for this damping mechanism is obvious given the sensitivity of cochlear hair cells to detect mechanical signals. The most direct blood supply to the organ of Corti is sparse, typically a small arteriole running adjacent to the basilar membrane. This may be one adaptation to keep pulsating vessels away from hair cells. On the other hand, the dense capillary network of the stria vascularis is richly supplied, and it is these feeding vessels that are described and modeled in the present study.

In many peripheral capillary beds (e.g., skin, lips, and retina), cardiovascular pulsation can be measured [1–3]. Blood pressures changes of up to 12 mmHg have been measured in the skin of healthy human adults [4]. This degree of pulsation in vessels of the stria vascularis would be highly disruptive to cochlear sensory mechanisms. At best, there would be a constant percept of heartbeat that might mask other auditory signals. More importantly, direct low-frequency pressure changes to the organ of Corti complex could amplitude modulating the whole system. Mechanical events could be of a magnitude to cause damage (an internal acoustic trauma). The present study explores the physical properties of vascular supply to stria vascularis and the adaptation of this system to attenuate cardiac pulse transmission. 

The vascular anatomy of the cochlea is examined using corrosion cast techniques. The injection of nonpolymerized plastic into the vasculature followed by polymerization, and “corrosion” of soft tissue, yields a cast of the interior lumen of the blood vessels. Such corrosion casts are imaged using scanning electron microscopy. In the present study, cochlear vasculature of the pig and the chinchilla are described. In particular, we examine the structure and function of tortuous arterioles that feed the capillary beds of stria vascularis. We have characterized some of the important properties of these vessels and we suggest that these convoluted vessels are adaptations to dampen the cardiovascular pressure changes that could disturb cochlear mechanics. We also present a mathematical model to illustrate the role of these vessels in attenuating cardiovascular pulsations within the cochlea. 

## 2. Materials and Methods

### 2.1. Corrosion Casting

Young adult pigs (*Sus scrofa; N* = 5) and adult chinchillas (*Chinchilla laniger; N* = 6) were used. In the anesthetized animal, a transcardial [5, 6] or aortic perfusion [7] of the head and upper body was carried out, clamping off lower body vessels. The right atrium was incised to allow venous return of perfusate. Perfusions were carried out using a peristaltic pump (Masterflex L/S Precision Variable-Drive peristaltic pump; Cole-Parmer Canada Inc., Montreal, QC, Canada) with a constant pressure of 100–140 mmHg. This pressure is in the normal physiological range so as to avoid possible distortion of the vessel architecture [8–13]. Initial perfusion was with heparinized (25000 U/L) [12] 0.9% NaCl (saline) to wash out all the blood and help to prevent blood clots forming in smaller capillaries. The saline was replaced by either Mercox II resin (Ladd Research, Williston, VT, USA) or Batsons' resin (Polysciences' Inc., Warrington, Pennsylvania, USA) for 1–3 minutes. Polymerization began 6–10 minutes after mixing the catalyst with the polymer. Once the resin hardened, the bulla/temporal bone was dissected and the cochlea exposed. This was then immersed in a 16% KOH solution over 3 days followed by 2% HCl (over 1 to 5 hours) to dissolve the soft tissue and the bone of the cochlea. The last step was the immersion of the sample in 16% KOH for another day before it was washed with distilled water to remove the remaining tissue. 

### 2.2. Scanning Electron Microscopy

Corrosion cast specimens were freeze dried [9, 14, 15] using a Micro-Modulyo freeze drying system (Thermo Fischer Scientific Inc.) and then mounted on a scanning EM stub. The samples where gold sputtered and analyzed for SEM with the Hitachi 3400 (Hitachi, Ltd., Chiyoda-ku, Tokyo, Japan) using either secondary electrons at 5 kV or backscatter electrons at 15 kV. The images where processed with Photoshop CS (Adobe Systems Incorporated Inc.) and analyzed with ImageJ (U.S. National Institutes of Health, Bethesda, MD, USA) software.

## 3. Results

### 3.1. Anatomical Studies

**Figure 1:**
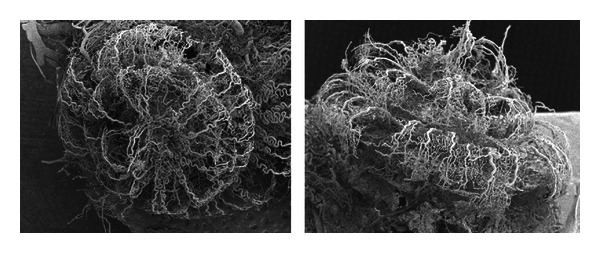
Scanning electron micrographs of (apical and lateral views) of the corrosion casts of cochlear vasculature in the pig.

**Figure 2:**
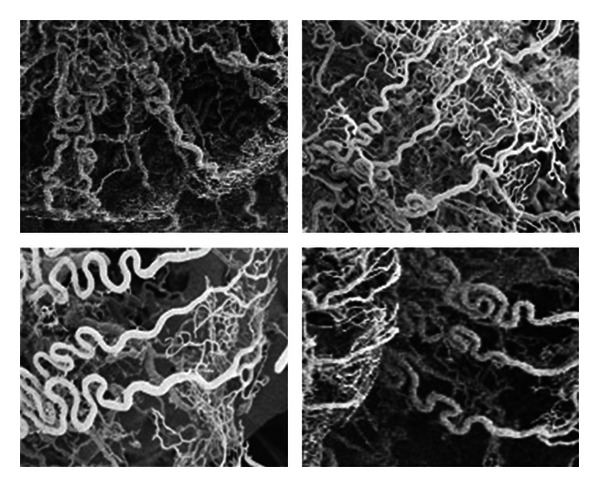
Tortuous arterioles that feed the stria vascularis (in pig), as seen in scanning electron images of corrosion cast specimens.

**Figure 3:**
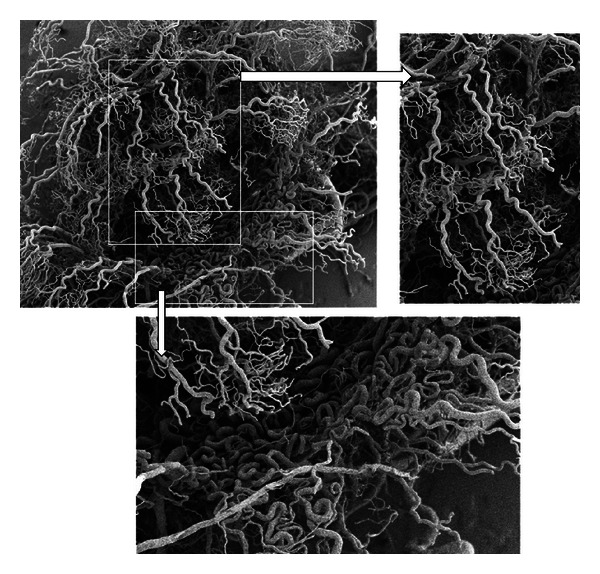
Partially filled corrosion casts of the pig cochlea showing only arterial blood supply vessels (capillary beds not filled). These images illustrate the extreme tortuosity in supply vessels to stria vascularis.

**Figure 4:**
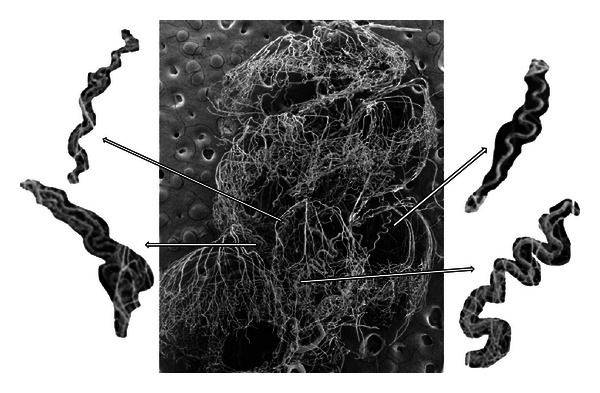
Scanning EM image of corrosion casts arterial and strial vessels of chinchilla cochlea. Note the convoluted arterioles that supply the stria vascularis.

**Figure 5:**
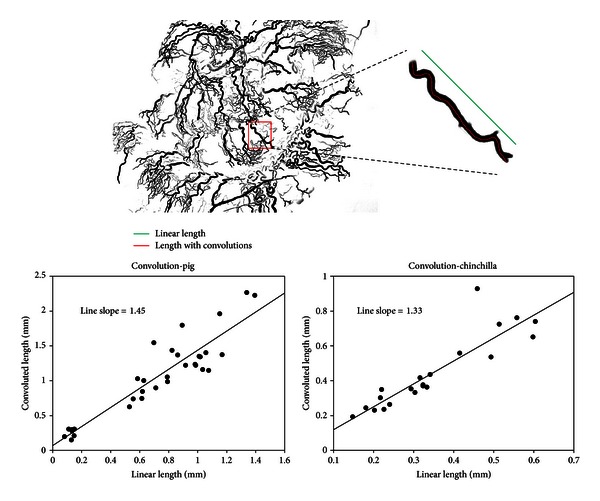
Method for quantifying the degree of arterial convolutions in vessels that supply the stria vascularis.

Representative images of corrosion casts of cochlear vasculature are shown in Figures 1–4. In Figure 1, from the pig, the overall shape of the cochlea is clearly evident, and its squat structure is different than that of the more elongated chinchilla cochlea of the shown in Figure 4. In most specimens, we attempt to fill all vasculature (arteries, capillaries, and veins), but in some cases a partial cast of only the arterial supply can be useful to more easily image some structures. The specimens of Figures 3–5 are partially filled casts that allow visualization of the arterioles that feed the stria vascularis. Note that these casts do not show the actual blood vessel themselves, but rather the interior lumen of the vessels.

Samples of the convoluted arterioles that supply the stria vascularis of the pig are shown in Figures 2 and 3. The tortuosity of these vessels is very distinctive. Note particularly in Figure 3 (lower panel) the structure of these vessels, which have been previously described as “cochlear glomeruli.” Figure 4 shows a corrosion cast from a chinchilla cochlea. In this case, the arterial supply and strial capillary beds and filled but not the veins. Some of the convoluted arterioles that supply the stria vascularis are highlighted. The degree of convolution in this species is not as great as in the pig. 

We have devised a simple method to quantify the degree of convolution in these vessels as illustrated in Figure 5. A measurement of the convoluted length divided by the linear end-to-end length provides a convolution index. In Figure 5, an example of such measurements form the pig vasculature is shown. The data graphs plot individual measurements from the pig (left plot) and from the chinchilla (right). The average convolution index can be noted from the slope of the linear regression drawn through the data sets. For the pig, on average there is higher tortuosity (1.45) compared with the chinchilla (1.33). We have noted differences in the degree of arteriole convolution when comparing basal and apical cochlear regions within species, but we have not yet made a systematic study.

## 4. Mathematical Model of Tortuous Vessels

**Figure 6:**
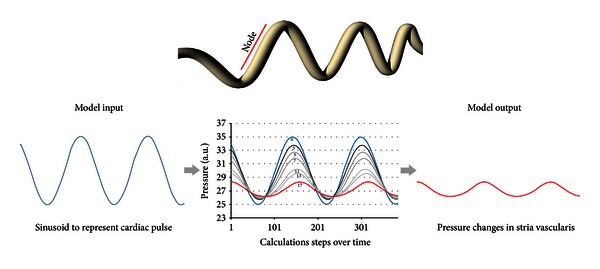
Model of a convoluted tube showing the definition of a node, that is, the section that precedes a bend in the tube. The lower panel shows the graphical representation of the mathematical model. The sinusoidal input (left) represents cardiovascular pulse signal into a convoluted vessel. The model output values are graphed (center plot) according to the number of nodes in the model. The model output (right) shows the attenuated pulse signal.

The arterioles feeding stria vascularis show a high degree of tortuosity, and we suppose that this physical structure can act to dampen (or low-pass filter) blood pulsations in the capillaries of the cochlea. We have developed a straightforward mathematical model to account for the damping that can be achieved by convoluted arterioles. The model describes the properties of an elastic walled tube having a series of convolutions (bends) as represented in Figure 6. Some basic assumptions are made which are only approximations to the actual blood vessels. Thus, the tube diameter is constant; we assume a laminar flow of a Newtonian liquid of undefined viscosity, and we make the assumption that liquid can only move forward in one direction. 

The resistance to fluid flow through the tube will in part depend on its length, and the convolutions provide a way of increasing the effective length of the tubes within the confined space of the cochlea. As shown in Figure 5, the lengths are increased from a factor of 1.33 (chinchilla) to 1.45 (pig) because of convolutions. In addition, each bend in a tortuous vessel will further increase the resistance to flow, especially in very small diameter tubes such as arterioles. Importantly in this model the tube wall acts as an elastic solid, and this basic property has been chosen based on the known viscoelastic properties of small arterioles. In this regard, Franz and colleagues [16] have reported that the second-order arterioles in the cochlea of the guinea pig have two smooth muscle layers that are known to have viscoelastic properties [17]. The third-order arterioles feeding the stria have little or no smooth muscle but have vascular pericytes cells that envelope the vessels that also have some intrinsic elastic properties [16].

Our model is based on a system with such viscoelastic properties. In a straight, elastic-walled tube, a sinusoidal pressure change would cause an overall volume change due to the compliant walls. However, in a convoluted, small diameter, elastic-walled vessel, each bend would add a resistance to flow such that tube segments before each bend would have increased viscoelastic distension. This is equivalent to separating the tube into a number of nodes (see Figure 6). For each node, not only is there a reduction in overall pressure change between input and output, but also each node acts as a pressure reservoir, and there is a consequent phase change to the pressure signal output from each node. The overall output from this system with many nodes (i.e., an arteriole with many convolutions) is the summation and a degree of cancellation of out-of-phase signals.

Assuming that the vessels are elastic, overall fluid dynamics will be the result of a competitive relationship among nodes. Each node is defined as the section of the vessel in front of a bend as illustrated in Figure 6. Equation (1) describes the elasticity and resistance of each node:

(1) $$\begin{array}{c} \frac{dN}{dt}=N\left(n-1\right)+N\left(n\right)-f\left(x\right)*N\left(n\right)-K. \end{array}$$

Here *N* is the pressure in any node, *f*(*x*) is a function that describes the elasticity of the tube, and *K* a constant that represents the resistance due to tube. Consider

(2) $$\begin{array}{c} f\left(x\right)=\frac{\alpha *N^{\delta }}{\left(\beta^{\delta }\right)+\left(N^{\delta }\right)}. \end{array}$$

We have modeled the tube wall elasticity based on known stress-strain curve data for an artery [18]. Thus in (2), properties of the elastic vessel walls are defined, where *α* is the maximal distension of the vessel, *β* is the value at which the elasticity of the walls reached the mid value (*f*(*x*) = *α*/2), and *δ* is the exponential value that describes the speed (time constant) at which the vessel returns at its initial position. 

Rather than a complex cardiovascular pressure waveform, we use a simple sinusoidal function (3) to simulate pulsatile blood flow:

(3) $$\begin{array}{c} \gamma *\sin\left(x*\sigma \right)+a. \end{array}$$

Here, *γ* is the pulsation amplitude of *f*(*x*) = sin, *σ* (angular frequency) represents heart rate, and *a* represents the average pressure (the average function position in the *y*-axis). Although the heart beat has a complex function, for our purposes it can be approximated as a sinusoidal function. The resultant model is shown below, where every *N*, except the first and the last, represents a node:

(4) $$\begin{array}{c} \frac{dN0}{dt}=\gamma *\sin\left(x*\sigma \right)+a,\\ \frac{dN1}{dt}=N0+N1-N1*\frac{\alpha *{N1}^{\delta }}{\left(\beta^{\delta }\right)+\left({N1}^{\delta }\right)},\\ \vdots \\ \frac{dN\left(n\right)}{dt}=N\left(n-1\right)+N\left(n\right)-N\left(n\right)*\frac{\alpha *{N\left(n\right)}^{\delta }}{\left(\beta^{\delta }\right)+\left({N\left(n\right)}^{\delta }\right)}-K. \end{array}$$

The graphical representation of this model is shown in the lower panel of Figure 6. The sinusoidal signal input (left) is unchanged in the model if there are zero nodes. Increasing node number (increasing bends in a convoluted blood vessel) clearly attenuates the transmitted pressure changes.

## 5. Discussion

In the present study, we used a corrosion casting technique and SEM imaging in order to assess the vasculature within the cochlea of chinchilla and pig. A number of previous studies have employed such methods to visualize and describe in detail the cochlear vasculature in various mammalian [16, 19–22] and avian [11] species. We present results from the pig *(Sus scrofa) *and chinchillas (*Chinchilla laniger*), species not previously investigated in detail. As described by others, we, observe the tortuous vessels that supply the capillary beds of stria vascularis. Tange [22] described corrosion casts of the adult rat inner ear and noted that apical and basal cochlear regions were supplied by the arteria cochleae propia and the arteria vestibulocochlearis, respectively. Images from that study showed convolutions of feeding arterioles but not a detailed description. In the guinea pig, Franz and colleagues [16] described the feeding vessels to stria vascularis as the “cochlear glomeruli,” as Schwalbe previously described them over a century ago [23]. These tortuous vessels are very similar to those found in the pig cochlea of the present study (see lower panel of Figure 3 in particular). These authors suggested that these tortuous vessels served to “reduce blood pressure by mechanical friction.” Others (e.g., [13, 20, 24]) have also observed these contorted vessels and generally suggested a role for blood flow regulation. Of interest, Iwagaki and colleagues [21] have studied age-related changes to cochlear vessels in mice and observe that in the neonate, vessels of the spiral ligament are less tortuous than in more mature animals. In this altricious species final cochlear development occurs some time after birth and it appears that the increased vessel convolutions might correspond with hearing onset and thus have some functional significance. 

We strongly suggest that the vessel convolutions serve to attenuate cardiovascular pulse signals reaching the capillary beds stria vascularis. In most peripheral capillary beds, a cardiac related pulse can be detected, and in some cases experimentally measured. Thus, Shore reported [4, 25] that in the peripheral capillary beds of the human finger there are cardiovascular pulse signals ranging from 0.5 mmHg (67 Pa) to 12 mmHg (1560 Pa). She used a direct cannulation of the capillaries with glass micropipette. For almost all capillary networks, it is of no detriment to have some pulsatile flow; however, in the stria vascularis of the cochlea it would pose a real problem. If in stria vascularis there were pulse pressure fluctuation at the lower end of the range reported for skin capillaries, for example, 0.5 mmHg (67 Pa) and if this was transmitted into cochlear endolymphatic space it would certainly interfere with cochlear function. It is unclear exactly how a low-frequency pulsation would affect hair cell function, but it is suffice to note that detection hair cells can detect acoustic signals of 20 *μ*Pa (0 dB SPL). A constantly pulsing pressure signal, many orders of magnitude larger than 20 *μ*Pa, would be a problem.

The mathematical model presented here is intended to simply illustrate how a tortuous and elastic vessel can attenuate pulsating pressure signals. As previously discussed, the model is relatively simple and does not incorporate some features that would clearly align it more closely with the actual vascular system. The exact shapes and dimensions of the arterioles are not modeled, nor is the non-Newtonian nature of blood and its possible laminar or turbulent flow characteristics. These elaborations can follow. The present model importantly serves as a valuable proof of principle. 

## 6. Conclusions

Using corrosion cast techniques, we have described the tortuous arterioles that supply the capillary beds of stria vascularis. We suggest that such vessel structure has an important role in dampening cardiovascular pulse signals that would otherwise interfere with cochlear function. We have presented a mathematical model to demonstrate the essential elements of this cardiac pulse attenuation system.
